# All the Sporting News

**Published:** 1907-04

**Authors:** 


### ALL THE SPORTING NEWS.

The steady reliability with which The Chicago Record-Herald presents the sporting news of the whole country each day has made it an authority in this field. It has a large staff of editors and reporters to handle the telegraphic matter and gather the local news in this important department alone. In The Sunday Record-Herald this department has a separate supplement of four full pages—a newspaper in itself—in which all the latest sporting news is presented with numerous illustrations. All that is of interest in baseball, football, racing, athletics, automobiling, yatching, golf, tennis, pugilism, aquatics and other sports can always be found here, along with many special articles by experts of national reputation. The Record-Herald is a favorite among lovers of sport because its sporting columns are written by "men who know."

